# Distinct contributions of frontal areas to emotion and social behaviour in the rat

**DOI:** 10.1111/j.1460-9568.2007.05844.x

**Published:** 2007-10

**Authors:** Peter H Rudebeck, Mark E Walton, Benjamin H P Millette, Elizabeth Shirley, Matthew F S Rushworth, David M Bannerman

**Affiliations:** Department of Experimental Psychology, University of Oxford South Parks Road, Oxford OX1 3UD, UK

**Keywords:** anxiety, behaviour, emotion, rat

## Abstract

Although the lesions of patients with impaired social behaviour encompass both orbitofrontal and anterior cingulate cortex (OFC and ACC), attempts to model such impairments in animals have focused on the OFC. However, recent neuroimaging attempts to identify the neural correlates of social interaction have emphasized the relative importance of ACC. Here we report the effect of circumscribed excitotoxic lesions of either OFC or ACC on ethological, unconditioned tests of emotion and social behaviour in the Lister hooded rat. OFC lesions altered emotional responsiveness to stimuli in non-social, fear-inducing situations (hyponeophagia test), and produced a small but statistically significant increase in aggression to other rats, but did not compromise other aspects of social interaction and appraisal. ACC lesions did, however, affect the utilization of social information. Specifically, ACC lesions diminished interest in other individuals and caused a relative reduction in memory for social stimuli. Whereas normal animals habituated to repeated presentations of the same individual, the poor performance of ACC animals entailed continued higher levels of responsiveness to repeated presentations of the same individual. The ACC impairment cannot simply be attributed to a general reduction in arousal, or a general impairment in recognition memory. Neither lesion affected anxiety per se (successive alleys test). Further analyses were conducted to investigate whether the changes in aggressive and social behaviour were related to different aspects of decision-making. Although the relationship between changes in social interaction and decision-making after ACC lesions is unclear, OFC impairments in emotionality were correlated with increased impulsive choice.

## Introduction

Attempts to model the changes in human social behaviour that follow damage to the ventromedial frontal lobe have mainly focused on the macaque orbitofrontal cortex (OFC; Bechara *et al.*, 2000). Studies consistently find that OFC lesions alter emotional responses to stimuli that induce mild fear (Butter *et al.*, 1970; Izquierdo *et al.*, 2005; Rudebeck *et al.*, 2006a), and it has been suggested that this emotional deficit is related to an inability to predict the reinforcing consequences of a stimulus (Rolls, 1999; Izquierdo *et al.*, 2005). However, frontal lesions that alter social behaviour in human patients are not just restricted to the OFC, but also extend medially into the anterior cingulate cortex (ACC).

Although it is agreed that normal emotional responsiveness is a prerequisite for normal social interaction, a number of recent neuroimaging studies that have directly assessed social interaction and evaluation have emphasized the importance of the ACC rather than the OFC (Amodio & Frith, 2006). In addition, one recent lesion study in macaques suggested complimentary roles for the OFC and ACC in mediating emotional responses and social interaction, respectively (Rudebeck *et al.*, 2006a). Lesions of the ACC gyrus, but not the OFC, altered the way in which social stimuli were valued, diminished interest in other macaques, and reduced affiliative behaviour. By contrast, lesions that included OFC made animals less responsive to mild fear-inducing stimuli.

While many investigations of ACC have employed neuroimaging methods, the lesion study conducted by Rudebeck and colleagues underlines the causal importance of the ACC in social interaction and evaluation (Rudebeck *et al.,* 2006a). Two important issues, however, remain unresolved.

First, it is not known if the changes in macaque social behaviour following discrete ACC lesions are also seen in other mammals after comparable lesions. Parts of the primate ACC affected by the lesion have homologues in the rat (Vogt & Peters, 1981). It is therefore important to investigate whether similar deficits in social and emotional behaviour will be observed in rats following lesions of similar areas.

Second, while OFC deficits in emotional responsiveness have been linked to impaired representation of reinforcement expectations (Rolls *et al.*, 1994), it is not clear if the role of the ACC in social interaction has a similar basis. There is evidence in the rat that OFC and ACC make dissociable contributions to decision-making and response selection (Mobini *et al.*, 2002; Walton *et al.*, 2003; Rudebeck *et al.*, 2006b). This dissociation raises the possibility that changes in emotion and/or social behaviour may be related to distinct decision-making processes. Alternatively, reduced social interest following ACC lesions could be the consequence of a general decrement in arousal, consistent with previous reports of ACC function (Neafsey *et al.*, 1993).

To investigate these issues, rats with either ACC or OFC lesions were tested on a number of previously validated ethological, unconditioned tests of social interaction and emotionality (Bannerman *et al.*, 2002b; McHugh *et al.*, 2004). Aspects of decision-making that depend on reward expectations had already been characterized in these subjects (Rudebeck *et al.*, 2006b). We also examined whether alterations in social behaviour were the result of a more general change in anxiety.

## Materials and methods

### Animals

Sixty-four Lister hooded rats (Harlan Olac, Bicester, UK) were used in the study. The study combined the results of two successive replications, which compared the effects of OFC, ACC and sham lesions. In replication 1, 34 animals were used, 11 OFC animals were compared with 11 ACC and 12 sham lesion animals. Replication 2 used 30 rats, seven OFC, 11 ACC and 12 sham lesion animals.

Animals were housed in cage groups of three, and maintained on a 12 h light : dark cycle. Training and testing always took place during the light phase. During the training and the testing phase all animals had access to food and water 24 h/day. Social behaviour and emotional responsiveness tests were conducted 5 months after lesions were made. Previously, animals had been preoperatively trained and then postoperatively tested on either a delay- (replication 1) or effort-based (replication 2) decision-making task (Rudebeck *et al.*, 2006b). All experiments were conducted in accordance with the UK Animals Scientific Procedures Act (1986).

### Surgical procedure

For replication 1, animals were anaesthetized with 1 mL/100 g Avertin (Avertin consists of 100 g of 2,2,2-tribomoethanol dissolved in 62 mL tertiary amyl alcohol; 1.25 mL of which is then added to 5 mL of absolute alcohol and 62.5 mL of 0.9% saline). For replication 2, animals were anaesthetized with isoflurane (Animal Care, York, UK). They were placed in a stereotaxic frame, and the head secured with bregma and lambda level. An incision was made along the midline and the area of bone above the target injection sites was removed. All injections were made using a 5-µL syringe with a specially adapted 34-gauge needle mounted onto the stereotaxic frame.

Based on previous reports (Chudasama & Robbins, 2003) and pilot studies in our lab, animals in the OFC lesion group received bilateral infusions of quinolinic acid (0.09 m) at the following three anterior-posterior (AP), mediolateral (ML) and dorsoventral (DV) coordinates: AP +4.0, ML ±0.8, DV −3.4 (0.15 µL); AP +3.7, ML ±2.0, DV −3.6 (0.2 µL); and AP +3.2, ML ±2.6, DV −4.4 (0.15 µL). AP and ML coordinates were measured relative to bregma, whereas DV coordinates were measured from the brain surface. ACC lesion rats received four bilateral infusions of quinolinic acid at: AP +2.3, ML ±0.5, DV −1.5 (0.2 µL); AP +1.6, ML ±0.5, DV −2.0 (0.2 µL); AP +0.9, ML ±0.5, DV −0.2 (0.2 µL); AP +0.2, ML ±0.5, DV −2.0 (0.2 µL). ACC lesion coordinates were the same as those used by Walton *et al.* (2003). For both lesions, quinolinic acid was infused at a rate of 0.1 µL every 30 s, with a 30-s interval between infusions. The needle remained in place for 3 min post-infusion to ensure the quinolinic acid diffused away from the injection site. Sham lesion surgery was conducted in the same way, although the needle was not lowered into the cortex. After all injections had been completed, animals were sutured and injected with an analgesic [Rimadyl®; 4 mg/kg (Carprofen), Pfizer, Sandwich, UK; or Metcam®; 0.3 mg/kg (Meloxicam), Boehringer Ingelheim, Germany for replications 1 and 2, respectively].

### Social memory

Two weeks before the social memory test adult animals were individually housed in transparent Perspex cages (45 × 24 × 20 cm). All subsequent testing was conducted in these cages. We closely followed the protocol we have used previously, which tested social memory at zero and 30 min delays, allowing comparison with other lesion studies (Bannerman *et al.*, 2001, 2002b). Juvenile stimulus animals (male Lister hooded, 35–65 g) were housed in groups of five in the same experimental room as the adult animals. On the day of testing juvenile animals were individually housed 1 h before the start of the test to isolate their odours. Adult animals in both replications 1 and 2 received three different social memory tests, conducted at least 48 h apart according to a fully counterbalanced design. The tests were: (i) a 30-min delay condition; (ii) a test during which a second, different juvenile animal was introduced to the adult after a 30-min delay; and (iii) a zero delay condition.

#### Thirty-min delay condition

A single juvenile animal was introduced into the cage of an adult for a period of 5 min. The interactions were recorded using a video camera mounted above the adult rat's home cage, which allowed off-line analysis of behaviour to be conducted. The amount of time that the adult spent investigating the younger animal was recorded by two investigators with stop clocks. Investigative behaviour was defined as the adult animal sniffing or grooming the younger animal, or closely following the conspecific as it moved around the cage. At the end of 5 min the juvenile was placed back in its holding cage (i.e. in isolation). After an interval of 30 min, the same juvenile was placed back in the adult's cage for a further 5-min period and the behaviour of the adult was observed. Social recognition memory manifests itself as a reduction in investigative behaviour between the first (T1) and second (T2) observation periods.

#### Different juvenile condition

Animals were tested in exactly the same way as the 30-min delay condition, except that during the second 5-min presentation period (T2) a different juvenile was placed in with the adult. As a result of a different juvenile being introduced a decrease in investigative behaviours between T1 and T2 would not be expected.

#### Zero delay condition

Similar to the two previous tests, a novel juvenile animal was placed into the cage of an adult animal and the amount of social investigation was recorded. Unlike the previous conditions the observation period lasted 10 min (T1 and T2 combined). As before the data were analysed in two 5-min blocks, T1 and T2.

#### Odour detection test

At the completion of social memory testing in replication 2, the olfactory abilities of the animals were assessed. The olfaction of animals in replication 1 was not assessed. Animals were exposed to two boxes containing soiled bedding from another adult animal's cage (novel odour) or soiled bedding from the adult's own cage (familiar odour) for a period of 5 min in their home cages. The position of the boxes in the cage was counterbalanced to control for any spatial bias. The amount of time each animal spent sniffing or investigating each box was recorded. Previous studies show that when presented with two odours sham lesion animals spent more time investigating the box containing the novel odour compared with the box containing the familiar odour (Bannerman *et al.*, 2001, 2002b).

### Adult social interaction test

Social interaction between two adult animals of the same lesion group (but not cage group) was assessed in a high-sided grey plastic box (35.5 cm long × 56.5 cm wide × 30.0 cm high) over a 5-min period. Both social and aggressive behaviours were recorded.

Unfamiliar testing environments have been shown to be anxiety provoking and lead to decreased social interaction in control animals (Shah & Treit, 2003). Therefore, to minimize the anxiety induced by the testing context, animals were habituated to the testing apparatus in cage mate groups (*n* = 3) for 5 min per day. Animals were considered habituated when no urine or faeces was left in the testing box at the end of the 5-min period.

On test days, two animals of the same lesion group that had never met before were brought into the test room in separate transport cages. On the day of testing an experimenter placed the two animals in the box facing away from each other and immediately left the room. Behaviour was monitored by means of a video camera and recorder for on- and off-line analysis. The durations of social and aggressive behaviours between the two animals during the 5-min period were recorded. Social behaviours were scored when animals engaged in anogenital investigation, sniffing each other, closely following each other and social grooming. Aggressive behaviours were scored when animals engaged in biting, upright boxing, aggressive mounting, chasing, induction of a submissive posture and aggressive grooming. Behaviour was independently scored by two separate observers, one of whom was blind to the lesion group assignment of each animal.

### Successive alleys test

The successive alleys test is a modified version of the elevated plus maze, and the apparatus has been described and validated previously (McHugh *et al.*, 2004). Briefly, the apparatus was made of four successive wooden alleys (sections 1–4) of increasing anxiogenic nature. The apparatus was mounted on the edge of a table 0.7 m above the floor, and was positioned such that section 1 was on the table whilst sections 2–4 extended out over the floor. Behaviour was monitored by means of a video camera mounted on the ceiling connected to a video recorder in an adjacent room allowing both on- and off-line analysis.

Animals were brought into the room 5 min before testing to standardize arousal levels. Animals were placed in section 1 of the apparatus facing away from sections 2–4. The animal's behaviour was recorded for 5 min. If a rat fell from the maze it was placed back on the apparatus at the junction between the section from which it fell and the previous section. The amount of time spent in each section, number of crossings into each section as well as the latency to cross from one section to another was recorded.

### Hyponeophagia

Hyponeophagia was assessed by measuring the latency to make contact with and eat a foodstuff in two potentially anxiogenic situations. The level of anxiety in each situation was manipulated by using both a novel foodstuff and by conducting the experiments in novel, potentially anxiogenic environments. Animals were food deprived for 24 h prior to testing. On the day of each test individual animals were removed from their home cages 5 min prior to testing and placed in a holding room adjacent to the testing room to standardize arousal levels. Animals were then brought into the testing room, placed in the apparatus, and the latency to: (i) make contact with; and (ii) eat the food were measured. If animals did not eat the food within 3 min they were removed from the apparatus and placed back in the holding room for a further 5 min before being retested. This procedure was repeated a second time if necessary, and if the animal still did not eat a score of 9 min/540 s was recorded. Each animal received two tests of hyponeophagia conducted at least 1 week apart. There were slight methodological differences between replications 1 and 2 with regards to the actual tests that were conducted. The precise methods were as follows.

#### Replication 1

##### Test 1

Latency to make contact with and eat pieces of sweetcorn were first recorded on a novel elevated T-maze (apparatus described elsewhere: McHugh *et al.*, 2004). Briefly, rats were placed on the T-maze facing away from the food well and the latencies to make contact with and eat the sweetcorn were measured.

##### Test 2

The second hyponeophagia test was conducted in a wire mesh box (61 cm long × 38 cm wide × 40 cm high) placed on the floor of a novel testing room. At the centre of the box was an aluminium food well in which a chocolate mini-button (Cadbury, UK) was placed. Rats were placed in the box facing away from the centre, and the latencies to make contact with and eat the chocolate were measured.

#### Replication 2

##### Test 1

The latencies to make contact with and eat a piece of sweetcorn were recorded on the same novel elevated T-maze as replication 1.

##### Test 2

The second hyponeophagia test measured the latencies to make contact with and eat a chocolate mini-button on a specially designed feeding table (apparatus described elsewhere: Bannerman *et al.*, 2002a). At the start of the experiment the sweetcorn and chocolate mini-buttons were novel to all the animals.

### Post mortem *lesion analysis*

At the conclusion of behavioural testing, animals were deeply anaesthetized with 200 mg/kg of sodium pentobarbitone (i.p.) and perfused transcardially with physiological saline, followed by 10% formalin saline. The brains were removed and stored in formalin saline solution. Subsequently, tissue was placed in sucrose–formalin solution for 24 h, frozen, sectioned coronally (25 µm) and stained with Cresyl violet. An experimenter unaware of the animal's behavioural performance assessed the extent of the lesions. Lesions are described with reference to Paxinos and Watson's stereotaxic rat brain atlas (Paxinos & Watson, 1998).

### Statistical analysis

Where possible, the data from all experiments were analysed using anova. In general, all analyses included a three-level between-subject factor of group (ACC, OFC, control) and replication (first and second experiment). Further *post hoc* Bonferroni tests or simple main effects analyses were conducted to explore any significant main effects or interactions resulting from the anovas. If the data failed the assumptions of normality or equal variance they were transformed (log10) to improve the distribution. However, if this failed to improve the distribution the data were analysed using a non-parametric Kruskal–Wallis test with further non-parametric independent sample Mann–Whitney U-tests to determine which groups were significantly different from each other.

## Results

### Histology

**Fig. 1:**
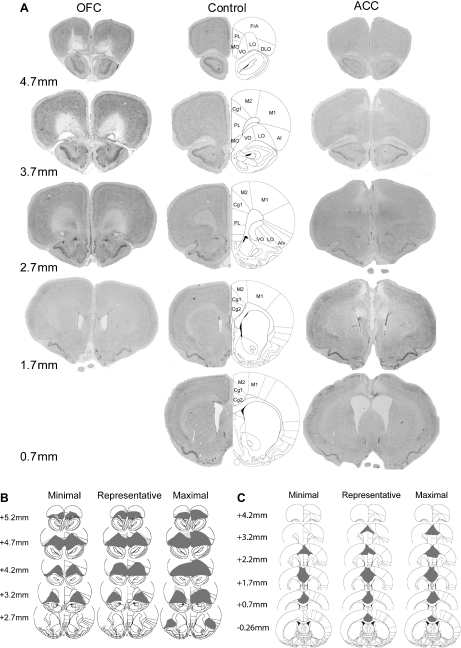
(A) Representative pictomicrographs of orbitofrontal cortex (OFC), anterior cingulate cortex (ACC) and sham lesions. Pictomicrographs of coronal sections (mm anterior to bregma) showing standard cell loss in representative OFC and ACC lesion animals compared with a sham lesion animal. Schematic coronal sections adapted from Paxinos & Watson (1998). AI, agranular insular cortex; AIv, agranular insular cortex ventral part; Cg1, cingulate cortex area 1; Cg2, cingulate area 2; DLO, dorsolateral orbital cortex; FrA, frontal association cortex; LO, lateral orbital cortex; M1, primary motor cortex; M2, secondary motor cortex; MO, medial orbital cortex; PL, prelimbic cortex; VO, ventral orbital cortex.(B) Reconstructions of the minimal (left), representative (centre) and maximal (right) OFC lesions. The size of the lesions in coronal sections between +5.2 mm and +2.2 mm anterior to bregma are illustrated.(C) Reconstructions of the minimal (left), representative (centre) and maximal (right) ACC lesions. The size of the lesions in coronal sections between +4.2 mm anterior to bregma and −0.26 mm posterior to bregma are illustrated. Dark shading represents areas of total cell loss. Light shading represents the lesion prenumba where cells were still present but were abnormal compared with those in sham lesion controls.

Both OFC and ACC lesions were highly selective, with no overlap between the lesions. OFC lesions reliably destroyed medial, ventral and lateral orbital cortex (Fig. 1A). In some of the smaller lesions there was some sparing of the most lateral areas of the dorsolateral orbital cortex. In all cases cell loss started approximately 5.2 mm anterior to bregma and extended as far back as 2.2–1.7 mm anterior to bregma (Fig. 1B). In a small number of animals, there was slight damage to the rostral infralimbic (IL) and prelimbic (PL) cortices between 4.7 and 3.2 mm anterior to bregma. The olfactory bulb was partially damaged in only a very small number of animals.

ACC lesions consistently and reproducibly destroyed both cingulate cortex areas 1 and 2 regions bilaterally (Fig. 1A) (Paxinos & Watson, 1998). The majority of lesions produced extensive cell loss in the ACC, starting approximately 3.2 mm anterior to bregma and extending back to about 0.26 mm posterior to bregma (Fig. 1C). In a small number of cases lesions extended further forward than 3.2 mm anterior of bregma, producing some damage in anterior PL. In some of the larger lesions there was also cell loss in the secondary motor cortex.

### Social memory

Data from one sham lesion animal were lost. Pearson correlation confirmed that there was good agreement between the two observers in all measures of social memory (*r* = 0.974; *P* < 0.001).

### Thirty-min delay condition

**Fig. 2:**
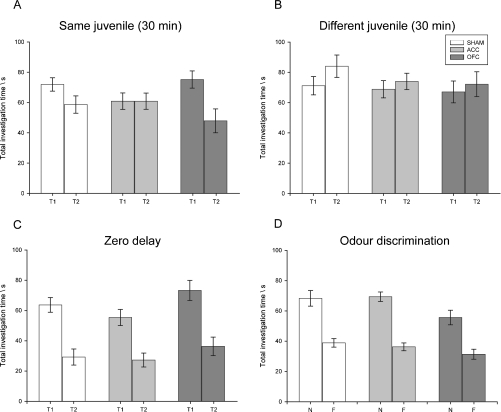
The effect of excitotoxic orbitofrontal cortex (OFC) or anterior cingulate cortex (ACC) lesions on social recognition memory in the rat. Mean (± SEM) total investigation time at first presentation (T1) and the second presentation (T2) 30 min later of either(A) the same juvenile or(B) a different juvenile.(C) Mean (± SEM) total investigation time when there was no delay between the first (T1) and second (T2) presentations of the same juvenile (zero delay condition).(D) Mean (± SEM) amount of time animals spent sniffing either a novel (different animal's bedding) or familiar odour (animal's own bedding).

Both OFC and sham lesion control animals showed a social memory effect, spending less time investigating the same juvenile animal at T2 compared with T1 (Fig. 2A). In contrast, animals with ACC lesions did not show the same decrease, and spent the same amount of time investigating the juvenile at T2 as T1. An anova of the total time animals spent investigating the juvenile at time T1 and T2 revealed no main effect of group, replication or group by replication interaction (*F* < 1; *P* > 0.5). However, there was a significant effect of time (T1 vs T2; *F*_1,57_ = 15.04; *P* < 0.001) and a group by time interaction (*F*_2,57_ = 4.89; *P* < 0.05). Further analysis of the group by time interaction using simple main effects revealed that there was a significant effect of time for both OFC (*F*_1,57_ = 17.37; *P* < 0.001) and sham lesion animals (*F*_1,57_ = 5.61; *P* < 0.05), but not for the ACC lesion group (*F*_1,57_ < 1; *P* > 0.5).

Next we calculated a difference score for each animal, the investigation time at T1 minus the investigation time at T2, for the 30-min delay condition. Analyses of the log_10_-transformed difference scores revealed a significant effect of group (*F*_2,47_ = 10.69; *P* < 0.01), and further *post hoc* Bonferroni tests showed that the difference score for the ACC lesion animals was significantly less than that of both OFC (*P* < 0.01) and sham lesion animals (*P* < 0.01), confirming that ACC lesion animals exhibited impaired social recognition memory.

It is possible, however, that the social recognition memory deficit was secondary to a deficit in social behaviour per se, and therefore the reason that ACC lesion animals did not show social recognition memory was that they simply spent less time investigating the juvenile at T1, which thus allowed them less opportunity to learn about the identity of the juvenile. A one-way anova comparing the amount of time all lesion groups spent investigating the juvenile at T1 suggested that this was not the case because there was not a significant group difference (*F*_2,62_ = 2.1, *P* > 0.1). Nevertheless, while not significantly different from OFC and sham lesion animals, it is still possible that the decreased investigation time of ACC animals at T1 might have caused a reduction in memory for the juvenile, and that social memory in these rats would be normal if the times spent engaged in social interaction with the juvenile during T1 were matched to controls. To test this possibility we conducted a further analysis.

**Fig. 3:**
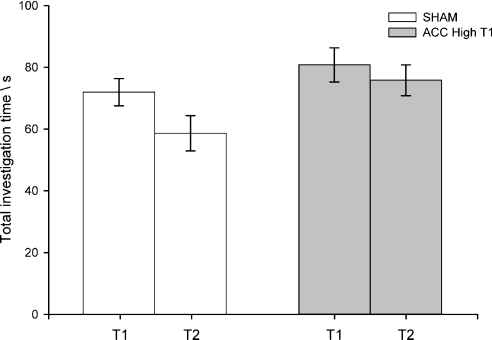
Anterior cingulate cortex (ACC) high T1 group compared with the sham lesion animals in the 30-min delay condition. Mean (± SEM) total investigation time at first presentation (T1) and the second presentation (T2) when the same juvenile animal was introduced 30 min later.

The ACC lesion group was divided on the basis of a median split into two subgroups, those with high (mean = 88.91 s) and those with low (mean = 48.73 s) investigation times at T1. This analysis was conducted to test whether a subpopulation of the ACC lesion group exhibiting high levels of investigation at T1, similar or higher than those of controls (T1 mean = 71.96 s), would show a social memory effect. An anova of the T1 and T2 times comparing high ACC T1 animals with controls revealed a main effect of group (*F*_1,32_ = 4.644; *P* < 0.05), but not an effect of time (*F*_1,32_ = 2.56; *P* > 0.1) or a group by time interaction (*F*_1,32_ = 1.36; *P* > 0.1); the high ACC T1 animals exhibited longer investigation times than controls at both T1 and T2 (Fig. 3). ACC animals spent more time investigating the juvenile at T2 (*t* = 2.233, d.f. = 32, *P* < 0.05) even when their initial investigation times at T1 were as long or longer than those of controls. Furthermore, as with the ACC group as a whole, the high ACC T1 animals did not show a social memory effect (main effect of time: *F*_1,10_ = 0.56; *P* > 0.1). In summary this additional analysis shows that the absence of a social memory effect in the ACC group cannot be attributed to decreased investigation at T1.

### Different juvenile condition

In contrast to the 30-min same juvenile condition, when a different juvenile was presented in the second exposure (T2) all animals showed slightly increased amounts of investigation at T2 compared with T1 (Fig. 2B). anova confirmed that there were no significant main effects of group, replication or an interaction between group and replication (*F* < 1; *P* > 0.1). However, there was a significant effect of time as all animals spent more time engaged investigating the second juvenile during T2 compared with the first juvenile at T1 (time: *F*_1,57_ = 5.45; *P* < 0.05). There was no group by time interaction (*F*_2,57_ < 1; *P* > 0.1), nor a group by replication by time interaction (*F*_2,57_ < 1; *P* > 0.5).

To investigate whether the ACC group treated the second presentation of the familiar conspecific as they would a novel juvenile animal, we compared the difference scores (T1 − T2) for the 30-min same juvenile condition (Fig. 2A) and the 30-min different juvenile condition (Fig. 2B) for all three groups. anova revealed a main effect of condition (novel vs familiar; *F*_1,60_ = 24.12; *P* < 0.001), and a lesion group by condition interaction (*F*_2,60_ = 3.61; *P* < 0.05). There was no significant main effect of lesion group (*F*_2,60_ = 2.28; *P* > 0.10). Subsequent analysis showed that whereas both the sham (*F*_1,22_ = 17.78; *P* < 0.001) and OFC (*F*_1,17_ = 11.22; *P* < 0.005) lesioned rats showed a significantly higher level of investigation for a novel juvenile compared with that for a familiar juvenile, this was not the case for the ACC lesioned rats (*F*_1,21_ < 1; *P* > 0.20). This analysis therefore suggests that ACC lesioned rats did not differentiate between novel and familiar conspecifics.

### Zero delay condition

Animals in all lesion groups showed a large reduction in the amount of time they spent investigating the juvenile in the second 5-min presentation period (T2; Fig. 2C). There was a significant effect of time (*F*_1,57_ = 100.74; *P* < 0.001), but no significant group by time interaction (*F*_2,57_ = 0.57; *P* > 0.5), as all animals spent a decreased amount of time investigating the juvenile at T2. There was no significant main effect of group (*F*_2,57_ = 1.73; *P* > 0.1), replication (*F*_1,57_ = 0.001; *P* > 0.5), or a group by replication interaction (*F*_2,57_ = 0.43; *P* > 0.1).

### Odour detection test

The odour detection test confirmed that all groups were able to distinguish between two different odours (Fig. 2D). anova confirmed that there was a significant effect of odour (*F*_2,27_ = 163.6; *P* < 0.001), with animals spending more time investigating the novel compared with the familiar odour, but not an effect of group (*F*_2,27_ = 2.49; *P* > 0.1). There was, however, a trend for a group by odour interaction (*F*_2,27_ = 3.05; *P* = 0.064], but further simple main effects analysis and *post hoc* Bonferroni tests failed to find any differences between groups. Simple main effects analysis further confirmed that all groups spent significantly more time investigating the novel odour (OFC: *F*_1,6_ = 39.62; *P* < 0.001; ACC: *F*_1,10_ = 90.54; *P* < 0.001; sham: *F*_1,11_ = 53.47; *P* < 0.001).

### Adult social interaction

**Fig. 4:**
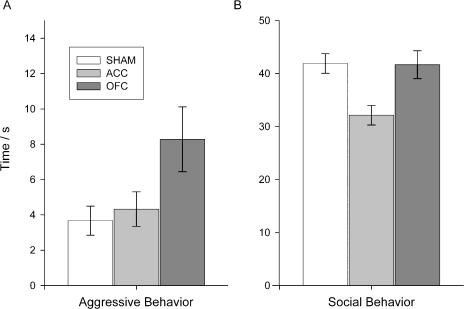
The effect of excitotoxic orbitofrontal cortex (OFC) or anterior cingulate cortex (ACC) lesions on social behaviour in the rat. The total amount of time animals spent engaged in either aggressive (A, mean ± SEM) or social behaviours (B, mean ± SEM) during the 300-s social interaction test.

ACC lesions decreased the amount of time spent engaged in social interaction between two adult rats, but had no significant effect on aggression (Fig. 4). Conversely, animals with OFC damage exhibited a small increase in aggression, but showed no changes in the amount of social interaction. Pearson's correlations conducted on the 162 separate interaction tests scored (43 OFC, 57 ACC and 62 sham) confirmed interobserver reliability for both social behaviours (*r* = 0.9, *P* < 0.001) and aggressive behaviours (*r* = 0.84, *P* < 0.001).

anova of the log_10_-transformed data for time spent engaging in social behaviours revealed a significant effect of group (*F*_2,156_ = 9.51; *P* < 0.001) but not replication (*F*_1,156_ = 0.9; *P* > 0.1), or a group by replication interaction (*F*_2,156_ = 1.67; *P* > 0.1). Further *post hoc* Bonferroni tests revealed that ACC lesion animals spent significantly less time engaging in social interaction than both OFC and sham lesion animals (*P* < 0.001). There was no difference between the amount of time OFC and sham lesion groups spent engaging in social interaction (*P* = 1).

The amount of time animals spent engaged in aggressive behaviours was very low by comparison with the amount of time animals spent socially interacting. The data were analysed using anova, which showed a significant effect of group (*F*_2,156_ = 7.068; *P* < 0.001). Bonferroni tests comparing the amount of aggressive behaviour between lesion groups confirmed that OFC lesion animals were significantly more aggressive than both ACC (*P* < 0.05) and sham lesion animals (*P* < 0.01). Importantly, there was no group by replication interaction (*F*_1,156_ = 0.56; *P* > 0.5). However, there was a main effect of replication (*F*_1,156_ = 36.71; *P* < 0.001). Therefore, some caution must be observed when interpreting these data. In addition, a non-parametric Kruskal–Wallis test on ranks was conducted and revealed a significant effect of group (*H*_2_ = 8.12; *P* < 0.05). Further analysis using Mann–Whitney tests showed that the OFC lesion animals were significantly more aggressive than both the ACC and sham lesion groups (*P* < 0.05; two-tailed). There was no difference between ACC and sham lesion groups (*P* > 0.1). In a further set of analyses, one-sample *t*-tests were conducted. The amount of aggressive behaviour that all groups engaged in was, although small, always significantly greater than zero (OFC: *t* = 4.513, d.f. = 42, *P* < 0.001; ACC: *t* = 4.426, d.f. = 56, *P* < 0.001; sham: *t* = 4.46, d.f. = 61, *P* < 0.001).

### The relationship between social behaviour and decision-making

To assess whether aggressive and social behaviours were related to different aspects of decision-making, two further sets of analyses were conducted. Previously, the animals in the present study had been trained preoperatively, and tested postoperatively, on a T-maze decision-making task (Rudebeck *et al.*, 2006b). The rats were trained in an enclosed T-maze to associate one arm of the maze with a high reward (HR arm, e.g. the left goal arm), and the other arm of the maze with a low reward (LR arm, e.g. the right goal arm). A cost was then introduced into the HR arm. In replication 1, rats were trained and tested on a delay-based decision-making task in which animals chose between waiting 15 s for the high reward or immediately obtaining the low reward. If the animals chose the high reward option, they were contained in the HR arm by a set of doors for 15 s prior to receiving the reward. In contrast, the animals in replication 2 were tested on an effort-based decision-making task, which assesses how much physical effort animals are prepared to expend to gain reward (Rudebeck *et al.*, 2006b). In this task rats chose between climbing a 30-cm wire mesh barrier to gain the high reward or expending no additional effort to gain the low reward.

**Fig. 5:**
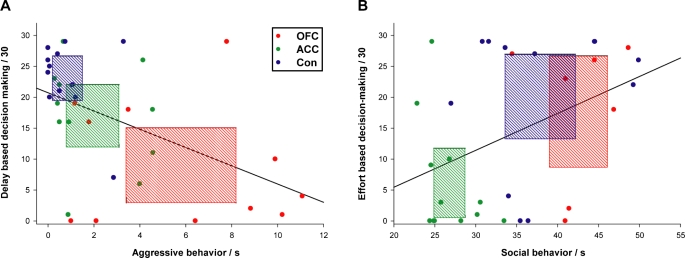
The relationship between: (A) aggressive behaviour and delay-based decision-making in the animals in replication 1; and (B) social behaviour and effort-based decision-making in the animals in replication 2.(A) Each coloured dot represents a single animal's aggressive behaviour score (amount of time in seconds) and performance on a delay-based decision-making task (number of HR arm choices, Block C, Experiment 1; Rudebeck *et al.*, 2006b).(B) Each coloured dot represents a single animal's social behaviour score (amount of time in seconds) and performance on an effort-based decision-making task (number of HR arm choices, Block C, Experiment 2; Rudebeck *et al.*, 2006b). Large coloured boxes represent two standard errors of the mean for the different lesion groups. A line of best fit has also been added. ACC, anterior cingulate cortex; OFC, orbitofrontal cortex.

First, the relationship between aggressive behaviour and impulsive choice, as measured by the number of HR arm choices made immediately post-lesion (Rudebeck *et al.*, 2006b; Experiment 1, Block C) on the delay-based decision-making task, was assessed (Fig. 5A). A significant negative correlation between these two measures would suggest that higher levels of aggressive behaviour may be related to increased impulsive choice. Separate analyses, conducted for each lesion condition individually, failed to find a significant correlation between these two measures within any of the three groups (Pearson's correlations, all *P* > 0.20). Subsequent power analyses revealed that there were not sufficient numbers in any of the groups to find a significant correlation. In contrast, however, a Pearson's correlation analysis conducted across all the animals in the study, irrespective of lesion condition, confirmed that there was a significant negative correlation between the number of HR arm choices in Block C of the delay-based decision-making task and the amount of time spent aggressively interacting (*r* = – 0.489, *n* = 34, *P* < 0.005; Fig. 5A). To ensure that the significant correlation was not simply caused by the performance of the OFC lesion group across both tasks, a similar analysis was conducted for just the sham operated and ACC lesion animals. Importantly, levels of aggressive behaviour and choices in the delay-based decision-making task were not significantly different between the ACC and control groups (*P* > 0.5). The Pearson's correlation revealed that there was still a significant negative correlation between aggressive behaviour and delay-based decision-making [*r* = – 0.357, *n* = 23, *P* < 0.05 (one-tailed test − there was a clear *a priori* prediction that increased aggression was correlated with increased impulsive choice, i.e. less HR arm choices)]. Thus, the significant correlation observed across all three groups is not simply due to the presence of the OFC group. Therefore, although these significant correlations arose from tests that were conducted by pooling data from two (or more) groups, and thus should be treated with some caution, they nevertheless provide initial evidence that there is a relationship between aggressive behaviour and impulsive choice.

To assess the relationship between social behaviour and effort-based decision-making, similar Pearson's correlations were conducted comparing the amount of social interaction and the number of HR arm choices animals made immediately post-lesion on the effort-based decision-making task (Rudebeck *et al.*, 2006b; Experiment 2, Block C; Fig. 5B). Again, individual analyses conducted separately for each group failed to reveal any significant correlations (*P* > 0.10). However, combining the data from all three groups did reveal a significant positive correlation between social interaction and animals performance on an effort-based decision-making task across all the animals in the study (*r* = 0.408, *n* = 31, *P* < 0.05). To determine whether or not this significant positive correlation was simply driven by the performance of the ACC lesion group across both tasks, a further analysis, which included both the OFC and sham lesion animals, was then conducted (importantly, these two groups did not differ in terms of social behaviour or effort-based decision-making). Pearson's correlation (*r* = 0.14, *n* = 19, *P* > 0.50) revealed that there was now no significant correlation between social behaviour and effort-based decision-making. The relationship between social interaction and effort-based decision-making therefore remains unclear.

### Successive alleys test

**Fig. 6:**
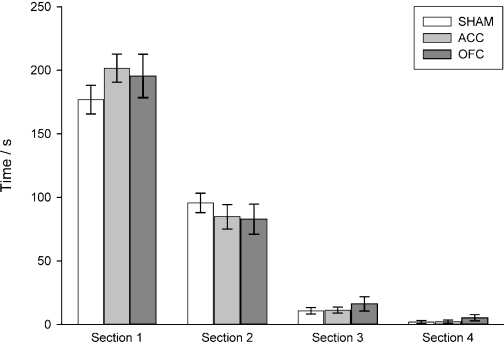
The effect of excitotoxic orbitofrontal cortex (OFC) or anterior cingulate cortex (ACC) lesions on the successive alleys test. Mean (± SEM) time spent in each of the four sections of the apparatus. Section 1 was the least anxiogenic in character, while section 4 was the most.

The data from three animals (one OFC, one ACC and one sham lesion animal) were lost due to a failure of the video recorder. All lesion groups showed similar levels of anxiety on the successive alleys test, spending the majority of the 5-min test in the first section/alley one and progressively less time in each of the other more anxiogenic sections (Fig. 6). There was no significant effect of replication (*F*_1,55_ = 1.43; *P* > 0.1) or a group by replication interaction (*F*_2,55_ = 1.55; *P* > 0.1). To correct for the fact that the time spent in each of the sections was not completely independent from each other, the numerator degrees of freedom were reduced for both the main effect of section and group by section interaction, and consequently the *P*-values were adjusted accordingly. An anova of the time the animals spent in each section revealed a significant main effect of section (*F*_2,165_ = 403.7; *P* < 0.001), but no group by section interaction (*F*_4,174_ = 1.62; *P* > 0.1), as all animals spent significantly more time in the less anxiogenic sections.

Analysis of the latency to enter each section and the total number of crossings between sections confirmed that the lesion groups behaved similarly. Non-parametric Kruskal–Wallis tests of the latency to enter sections 2, 3 and 4 revealed no differences between lesion groups (section 2: *H*_2_ = 1.53; *P* > 0.1; section 3: *H*_2_ = 2.06; *P* > 0.1; section 4: *H*_2_ = 1.67; *P* > 0.1). anova of the total number of crossings between the different sections similarly failed to show a significant difference between the groups (*F* < 1; *P* > 0.1).

### Hyponeophagia

**Fig. 7:**
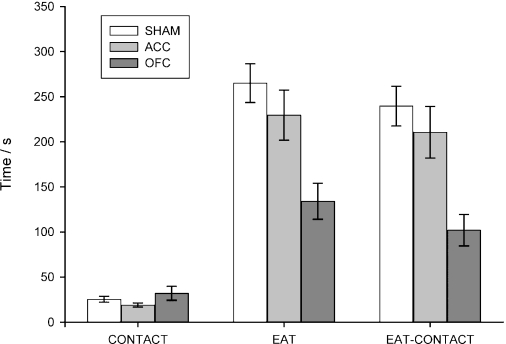
The effect of excitotoxic orbitofrontal cortex (OFC) or anterior cingulate cortex (ACC) lesions on hyponeophagia in the rat. Mean (± SEM) latency to make contact with a novel foodstuff, unambiguously start eating the novel foodstuff, and the difference between the latency to make contact with the food and begin eating the food.

The data from one ACC lesion animal was lost. Animals in all groups made contact with the novel food item after a similar amount of time (*F*_2,57_ = 1.902; *P* > 0.1). However, OFC lesion animals began eating earlier in the test period than both the ACC and sham lesion groups (Fig. 7). Analysis of the latency to begin eating minus the latency to make contact with the foodstuff revealed that there was no effect of replication (*F*_1,57_ < 1; *P* > 0.5), nor a group by replication interaction (*F*_2,57_ < 1; *P* > 0.5). Importantly, there was a significant main effect of group (*F*_2,57_ = 8.14; *P* < 0.001), regardless of the test format (absence of group by test interaction: *F*_2,57_ < 1; *P* > 0.5), and further *post hoc* Bonferroni tests confirmed that OFC lesion animals were significantly quicker to start eating compared with both ACC and sham lesion animals (*P* < 0.05). There was no difference between ACC and sham lesion animals (*P* > 0.1).

## Discussion

Frontal lesions altered animals' emotional responses and social behaviour; the patterns of changes after ACC or OFC lesions were distinct. OFC lesions were associated with altered emotional responsiveness in hyponeophagia and a small, but statistically significant increase in aggressive behaviour in the adult social interaction test. By contrast, ACC lesions reduced social behaviour in the social interaction test and impaired the utilization of social information in the social memory test. This dissociation suggests that distinct mechanisms, dependent on ACC and OFC, may mediate social interaction and emotional responses, respectively. Deficits in emotional responsiveness were correlated with changes in performance on a delay-based decision-making task (Rudebeck *et al.*, 2006b), suggesting that OFC-dependent emotional changes may be related to increased impulsive choice. Whether ACC lesion-related changes in the valuation of social information are related to aspects of impaired action valuation and decision-making, however, is not clear.

### OFC and emotional responsiveness

A number of reports have linked the OFC with emotional responsiveness in animals (Kolb, 1974; de Bruin *et al.*, 1983; Izquierdo *et al.*, 2005; Machado & Bachevalier, 2006), but two issues have remained unclear; whether the same lesions also affect social interaction and whether other frontal lesions cause similar effects. In the present study, OFC lesion animals valued the opportunity to acquire social information about other individuals as much as controls. OFC lesion and control animals engaged in similar amounts of social behaviours, such as sniffing and close following, on encountering an unfamiliar adult (Fig. 4) or juvenile (Fig. 2). Moreover, OFC animals exhibited normal patterns of social memory. In the social memory test rats had the opportunity to interact with juvenile rats on two occasions; sometimes the same juvenile twice, but on other tests a different juvenile on each occasion. Control and OFC groups showed less interest when the same juvenile was presented again, either immediately after the first encounter (Fig. 2C) or after delay (Fig. 2A), but they continued to engage in a high rate of socially orientated investigative behaviours when the identity of the juvenile was changed (Fig. 2B).

Although OFC lesions did not diminish animals' social engagement, they led to a small but significant increase in aggression (Fig. 4), and decreased fear in response to unconditioned stimuli on the hyponeophagia test (Fig. 7). It is important to note that the overall levels of aggression were low, and amounted to only 2% of the test duration, and thus some caution is required in interpreting these data. Furthermore, the significant main effect of replication observed for this test may also indicate that the prior history of the animals may have had an influence on the outcome. Importantly, however, there was no group by replication interaction, and there is no *a priori* reason for expecting that different histories on the T-maze would necessarily influence aggression levels. Indeed, closer inspection of the data reveals that the same pattern of results was observed across the three groups of rats, for both social and aggressive behaviours, in both replications of the study (i.e. irrespective of the nature of the prior maze testing; see Table 1).

**Table 1:** The effect of replication on the amount of time animals spent engaged in aggressive and social behaviours during the 300-s social interactions test

	Social interaction	
	
Group and replications	Aggression	Social
OFC		
1	5.86 ± 1.68	41.33 ± 3.33
2	16.255 ± 5.01	42.84 ± 3.05
ACC		
1	2.12 ± 0.82	34.63 ± 2.57
2	8.75 ± 1.51	27.16 ± 1.17
Sham		
1	0.875 ± 0.47	44.1 ± 2.48
2	8.77 ± 1.70	37.91 ± 2.39

Data are presented as means ±SEM. ACC, anterior cingulate cortex; OFC, orbitofrontal cortex.

Furthermore, previous lesion studies have also implicated the rat OFC in aggression (Kolb, 1974; de Bruin *et al.*, 1983). There is also a striking similarity in the changes in emotional responsivity following OFC lesions in rats and macaques. A small increase in aggression and a decrease in fear for unconditioned but ethologically significant stimuli is prominent when OFC lesions are made in the macaque (Izquierdo *et al.*, 2005; Machado & Bachevalier, 2006; Rudebeck *et al.*, 2006a). Following OFC lesions, macaques more readily take food from the top of a Perspex box containing fearful objects and, in other situations, they were more aggressive to unfamiliar humans (Izquierdo *et al.*, 2005). As in the current experiment the effect is specific to OFC; ACC lesions did not significantly alter fear or aggression in macaques. Furthermore, because the lesions in the current study were made by excitotoxin, in contrast to the non-fibre sparing approaches adopted in earlier studies, including the studies in primates (Kolb, 1974; de Bruin *et al.*, 1983; Izquierdo *et al.*, 2005; Rudebeck *et al.*, 2006a), it is unlikely that OFC lesion-induced changes in emotional responsiveness are the result of inadvertent damage to the underlying white matter.

These findings in macaques were interpreted as the consequence of an impairment in response selection, driven by the inability to represent expected outcomes (Izquierdo *et al.*, 2005). The propensity for OFC-lesioned rats to eat novel foods more quickly may similarly reflect a failure to properly integrate the anxiogenic nature of the novel environment with the potentially rewarding properties of the food leading to the production of an inappropriate or impulsive response of eating. All animals took a similar amount of time to make contact with the novel food item, but only the OFC lesion group had shorter latencies to begin eating, suggesting that they failed to integrate the potential cost associated with the novel environment and the potential value of the reward before acting.

The present study may thus provide further insight into why changes in emotional responsiveness are seen after OFC lesions. Importantly, it rules out the possibility that such changes are contingent on a general change in anxiety because none was observed in the adult social interaction or successive alleys tests. Instead, the present data may provide further support for the hypothesis that alterations in fear and aggression after OFC lesions may be related to an inability to generate outcome expectancies, as suggested by Izquierdo *et al.* (2005). A growing body of evidence suggests that the OFC is critical for representing the value of goals or outcomes (Baxter *et al.*, 2000; Bechara *et al.*, 2000; Izquierdo *et al.*, 2004; Roesch & Olson, 2004; Roesch *et al.*, 2006). We have argued previously that an inability to represent the expected value of outcomes may explain why rats chose impulsively in the delay-based decision-making task (Mobini *et al.*, 2002; Rudebeck *et al.*, 2006b). Some animals in the present study also performed the delay-based decision-making task. The same OFC lesion affected performance on this task as well as both fear and aggression levels, suggesting that they may share a common neuroanatomical basis.

Therefore, the OFC-related increase in aggression in the social interaction test, although small, may similarly be construed as the selection of inappropriate or impulsive choices when confronted by a novel stimulus, in this case another adult rat. The correlation analyses conducted (Fig. 5A) showed that the amount of aggressive behaviour exhibited by the animals in replication 1 was inversely correlated with their impulsive performance on a delay-based decision-making task, a task that is thought to require the ability to generate outcome expectations. Higher scores on the delay-based decision-making task (less impulsivity) are associated with lower levels of aggression, while a low score on the decision-making task (high impulsivity) was associated with heightened levels of aggression. However, further experiments are needed to clarify the relationship between outcome expectations and aggressive behaviour.

In a recent study, Ross and colleagues showed that rats with cholinergic depletions of the OFC were impaired in acquiring social transmission of food preference (STFP; Ross *et al.*, 2005). STFP describes the process whereby rats are more inclined to eat a novel foodstuff if they have previously engaged in social interaction with a conspecific that has just recently ingested that same foodstuff (Galef, 1986). It is thought that STFP might lessen the risk of eating novel (potentially toxic) foods, because a food recently eaten by a healthy animal is apparently safe. The observation from the present study that OFC lesions do not affect the amount of social behaviour, or the utilization of social information, suggests that the deficit in STFP with OFC lesions is not due to the social context of the experiment. Rather, the impairment in STFP is most likely due to an inability to associate a particular stimulus with a specific outcome, and thus reflects a deficit in generating an accurate outcome expectancy.

### The ACC and the utilization of social information

Although ACC lesions did not alter fear or aggression, they did disrupt the utilization of social information (Figs 2 and 4). The lowered valuation of social information after ACC lesions is strikingly similar to that seen when lesions of the ACC (Hadland *et al.*, 2003), and specifically the ACC gyrus (Rudebeck *et al.*, 2006a), are made in macaques. Normal male macaques value the opportunity to gain more information about a high status male or a female macaque, but this is not the case after ACC gyrus lesions. Like the rats with ACC lesions in the present experiment, macaques with ACC lesions spend less time in proximity with one another, but there is no consistent change in fear or aggression. The current results show that the absence of a change in fear and aggression reported after ACC lesions in these macaques was not simply a consequence of low statistical power.

The role of the ACC in social behaviour may also extend to humans. Neuroimaging studies have reported ACC activation when people perform tasks that require cooperation, such as the prisoner's dilemma game (Rilling *et al.*, 2002, 2004) and the trust game (King-Casas *et al.*, 2005; Tomlin *et al.*, 2006). Cooperation in such tasks is more stable when participants have the opportunity to find out about each other (Fehr & Fischbacher, 2003). The current results suggest that the ACC activation that occurs when participants make and monitor choices in cooperation games reflects the acquisition and retrieval of information about the other player. Other studies have reported ACC activation during social evaluation and theory of mind tasks that require consideration of other individuals (Amodio & Frith, 2006). Furthermore, lesions that include parts of the ACC and OFC are associated with social and emotional changes in patients (Cummings, 1993; Hornak *et al.*, 2003).

The present results underline the causal importance of the ACC for normal social engagement and for the utilization of social information. Successive alleys and hyponeophagia tests demonstrated that changes in social behaviour following ACC lesions are not the consequence of changes in anxiety (Figs 6 and 7). Moreover, unlike OFC lesions, the representation of expected outcomes in the delay-based decision-making task was not affected by ACC lesions (Rudebeck *et al.*, 2006b).

Anatomical studies and stimulation experiments have demonstrated that some regions of the ACC are important in autonomic control (Burns & Wyss, 1985; Floyd *et al.*, 2000; Gabbott *et al.*, 2005). It is difficult, however, to attribute the present finding of altered retention of social information after the ACC lesion to a general change in arousal. An account of the ACC lesion deficit couched in terms of a general decrease in arousal would also have predicted changes in behaviour on the successive alleys and hyponeophagia anxiety tests. On the contrary, however, ACC lesion animals showed intact anxiety and fear responses on these two tests, suggesting that aspects of arousal were unchanged. One explanation is that the ACC is important for regulating arousal specifically during social interactions. However, it is worth pointing out that whereas normal animals habituated to repeated presentations of the same individual in the social memory test, the poor performance of ACC animals actually involved maintaining higher levels of responding to repeated presentations of the same individual.

The deficit in social recognition memory is likely to reflect a specific deficit in the processing of social information rather than a general memory impairment. For example, previous studies have shown that lesions including the ACC do not affect object recognition memory (Ennaceur *et al.*, 1997; Yee, 2000). In the spontaneous object recognition memory task animals are commonly presented with two identical objects and then after a delay period are presented with a third identical object and a novel object. Object recognition memory manifests itself as an increase in investigation time of the novel, as opposed to the familiar, object. Animals with lesions of the ACC are unaffected on the spontaneous object recognition task and perform similarly to control animals (Ennaceur *et al.*, 1997; Yee, 2000). Both groups show a significant and equivalent preference for the novel object. Therefore, the deficit with ACC lesions appears to be specific to social situations and cannot simply be attributed to a disruption in mnemonic processing per se.

An alternative explanation is that altered social behaviour may be related to a different type of decision-making deficit. Although animals with ACC lesions are unimpaired on delay-based decision-making tasks, the ACC is critical for evaluation of how much effort it is worth investing to obtain a larger reward in rats (Walton *et al.*, 2003; Rudebeck *et al.*, 2006b). Studies in macaques have shown that ACC neurons represent an animals' progression through a work schedule (Shidara & Richmond, 2002), suggesting that the ACC may have a similar role in the primate brain. While such tasks may not assess the representation of future reinforcement or outcome expectations, they may instead probe a representation of the intrinsic value of actions that comprises both the action's costs and benefits. It is possible that there is a shared basis for the changes in action valuation and the changes in social valuation that are seen after ACC lesions. However, the correlation analyses performed as part of the present study, examining a potential relationship between performance on an effort-based decision-making task and the amount of time spent socially interacting, failed to fully support this hypothesis (Fig. 5B).

Indeed, evidence from macaques and humans suggests that deficits in decision-making and social behaviour following ACC lesions may not in fact share the same neural substrates. In macaques deficits in decision-making and social valuation are most closely associated with the ACC sulcus and gyrus, respectively (Kennerley *et al.*, 2006; Rudebeck *et al.*, 2006a). Similarly, human imaging studies have shown that dorsal ACC is activated following expectancy violations, whereas ventral ACC is more active during social feedback and memory retrieval (Mitchell *et al.*, 2004; Somerville *et al.*, 2006). This functional correspondence within the ACC between different species suggests that a region of the ACC specialized for social behaviour may be common to all mammals, including rats.

## Abbreviations

ACC: anterior cingulate cortex
AP: anterior-posterior
DV: dorsoventral
HR arm: high reward arm
IL: infralimbic
LR arm: low reward arm
ML: mediolateral
OFC: orbitofrontal cortex
PL: prelimbic
STFP: social transmission of food preference.
